# Alterations of Serum Biochemical and Urinary Parameters in a Canine Population before and after Intravenous Contrast Administration

**DOI:** 10.3390/vetsci8080146

**Published:** 2021-07-29

**Authors:** Federica Cagnasso, Barbara Bruno, Claudio Bellino, Antonio Borrelli, Ilaria Lippi, Barbara Miniscalco, Silvia Roncone, Alberto Valazza, Renato Zanatta, Paola Gianella

**Affiliations:** 1 Department of Veterinary Sciences, University of Turin, Largo P. Braccini 2, 10095 Grugliasco, Italy; federica.cagnasso@unito.it (F.C.); barbara.bruno@unito.it (B.B.); claudio.bellino@unito.it (C.B.); antonio.borrelli@unito.it (A.B.); barbara.miniscalco@unito.it (B.M.); milvia.roncone@gmail.com (S.R.); alberto.valazza@unito.it (A.V.); renato.zanatta@unito.it (R.Z.); 2 Department of Veterinary Sciences, University of Pisa, Via Livornese Lato Monte, 56121 Pisa, Italy; ilaria.lippi@unipi.it

**Keywords:** dog, iodinated contrast medium, symmetric dimethylarginine, alkaline phosphatase/creatinine ratio, γ-glutamyl transferase/creatinine ratio, urinalysis

## Abstract

Intravenous iodinated contrast (IVIC) medium is routinely administered to dogs. Scattered information exists regarding the serum biochemical or urinary profiles associated with the administration of IVIC in dogs. The aim of the study was to describe, compare, and discuss from the perspective of previous studies the alterations in serum biochemical and urinary parameters before (T0) and within one week (T1) of the IVIC administration during routine computed tomography (CT) scan evaluation of 22 dogs. Mature dogs presenting for CT scan evaluation for preoperative oncology staging/surgical planning were included. T1 evaluation was performed within one week of IVIC administration. Statistically significant differences in serum total protein, albumin, chloride, calcium, and phosphorus concentrations, urine protein to creatinine ratio, and urine specific gravity were found between T1 and T0. At T1, the serum creatinine concentration was within reference ranges in all dogs but one. An increase in the urine protein to creatinine ratio was observed in four samples, one of which was non-proteinuric at T0. Changes in biochemistry and urine parameters between T0 and T1 were not considered clinically significant.

## 1. Introduction

Iodinated contrast media are among the most frequently used contrast agents in medical practice [1]. However, mild to severe adverse reactions, particularly contrast-induced acute kidney injury (CI-AKI), have been observed in humans [2,3,4]. For years, in human medicine, CI-AKI has been defined as an increase in serum creatinine concentration by ≥0.5 mg/dL (44 µmol/L), or ≥25% from baseline, within 5 days from IVIC administration [5,6]. More recently, the 2011 KDIGO released guidelines with a new CI-AKI definition, namely an increase in serum creatinine concentration by ≥0.3 mg/dL (26.5 µmol/L) within 48 h, or an increase of ≥1.5 times baseline within 7 days of IVIC administration [7]. The decline in kidney function can range from mild to severe [8]. In the veterinary counterpart, the IRIS group has recommended a 5-grade system of disease severity for AKI, which is based on the serum creatinine concentration. However, it does not describe a steady-state condition, the renal function and the serum creatinine varying continuously in AKI [9]. Historically, the incidence of CI-AKI varied up to 50% in high-risk human patients [5,10,11,12]; however, more recently, a decreased incidence has been observed [13,14], probably due to better patient management or use of the safest contrast media. Common risk factors associated with CI-AKI in human medicine are diabetes mellitus, anemia, advanced age, nephrotoxic medications, cardiovascular diseases, and pre-existing kidney diseases, particularly advanced chronic kidney disease [5,11,12,15,16,17,18,19,20,21,22]. Indeed, an advanced chronic kidney disease contributes to a threefold increase in the risk of CI-AKI development [11].

In the veterinary counterpart, intravenous iodinated contrast (IVIC) medium is routinely administered to dogs undergoing advanced diagnostic imaging such as computed tomography, cardiac catheterization, intravenous pyelography, or angiography [19,20,21]. However, limited information exists regarding serum biochemical or urinary profiles associated with the administration of IVIC in dogs [22,23,24,25,26,27,28,29,30]. Moreover, a temporal association between the administration of IVIC and acute kidney injury has been scarcely demonstrated [28,30,31]; increases in serum creatinine concentration, low urine output, and/or acute oliguric renal failure were described in these studies within a short time of IVIC administration, highlighting the potential for CI-AKI in dogs. The small number of dogs evaluated, the delay between contrast administration and post-contrast evaluation of creatinine, the failure to use sensitive biomarkers for the recognition of early acute kidney injury, the lack of urinalysis evaluation, and the retrospective nature of some studies are the major limitations that may have impaired an accurate assessment of CI-AKI in dogs [28,29,30,31].

The goal of our study was to evaluate for changes in selected biochemical and urine parameters before and after IVIC administration for CT scanning performed under general anesthesia in dogs with neoplasia.

## 2. Materials and Methods

This study included 22 privately owned dogs undergoing routine IVIC administration during CT scan evaluation for preoperative oncologic staging/surgical planning. The population was prospectively enrolled, during the period from July 2019 to July 2020, from the Veterinary Teaching Hospital, Department of Veterinary Sciences, University of Turin. The protocol was approved by the Institutional Ethics and Animal Welfare Committee (approval number 664), and all owners gave informed consent.

Dogs were selected according to the following inclusion criteria: being older than 5 years, requiring a CT scan evaluation for preoperative oncologic staging/surgical planning, being unaffected by acute kidney injury, and being able to return for blood and urine reevaluation within 1 week following IVIC administration. AKI was excluded based on the International Renal Interest Society (IRIS) guidelines and grading system [9]. All available information, including serum creatinine concentrations before the study, urinalysis, and diagnostic imaging findings, was used. Dogs that had undergone anesthesia or received potentially nephrotoxic drugs within 2 weeks prior to selection were excluded, as well as dogs that had received intravenous fluids, medications known to induce proteinuria, and/or any other medication.

The first evaluation (T0) was carried out prior to fluid, anesthesia, and IVIC administration, on the same day as the CT scan evaluation and IVIC administration were performed. The second evaluation (T1) was carried out 3 to 7 days after IVIC administration and on the same day as the CT report was available for discussion.

All CT studies were performed under general anesthesia using a 16-slice CT scanner (Somatom Emotion, Siemens). A 5 min preoxygenation period was used. Intravenous propofol for induction and isoflurane mixed with oxygen and administered via a gaseous endotracheal tube for maintenance were used in all dogs. A bolus of 600 milligrams per kilogram body weight of the tri-iodinated non-ionic monomeric contrast medium iomeprol (300 mg/mL Iomeron for dogs ≤ 30 kg and 400 mg/mL Iomeron for dogs > 30 kg; Bracco, Imaging Italia s.r.l., Milano, Italy) was administered via the cephalic vein. Balanced isotonic crystalloid fluids (lactate Ringer solutions) were administered IV to all dogs at a maintenance rate of about 3–5 mL/kg/h. ECG, blood pressure, and pulse oximetry were constantly monitored.

Serum obtained from blood collected from each dog immediately before fluid, anesthesia, and IVIC administration (T0) and, after IVIC administration (T1), was sent to an external laboratory (Idexx Laboratories, Novara, Italy). The following serum biochemical parameters were evaluated: symmetric dimethylarginine (SDMA), creatinine, blood urea nitrogen, total protein, albumin, chloride, phosphorus, potassium, calcium, and sodium. Urine samples were collected immediately before anesthesia and IVIC administration (T0) and after IVIC administration (T1) by spontaneous micturition and analyzed within 30 min of collection. At T1, urine and blood were collected from each dog on the same day. The urine samples were analyzed by use of a urine dipstick (Clinitek Status+ Analyzer, Siemens, Italy). Five milliliters of each sample were centrifuged, and the urine sediment underwent microscopic examination. Fewer than 5 leukocytes and red blood cells per high-powered field were considered a normal finding. In addition, urine specific gravity (USG), urine protein to creatinine ratio (UPC), urinary γ-glutamyl transpeptidase (uGGT), urinary γ-glutamyl transpeptidase to urinary creatinine ratio (uGGT/uc), urinary alkaline phosphatase (uALP), and urinary alkaline phosphatase to urinary creatinine ratio (uALP/uc) were evaluated. Urinary biochemistry was determined using a wet chemistry analyzer (BT 3500 VET, Futurlab, Italy). Enzymatic activity was expressed in units per liter (U/L). Urine specific gravity was determined using a manual handheld refractometer (Reichert Vet 360, TS Meter Model). Based on the substaging system of the International Renal Interest Society (IRIS), borderline proteinuria was defined as having a UPC ≥ 0.2 to ≤0.5, and proteinuria was defined as having a UPC > 0.5. Samples with a UPC < 0.2 were considered non-proteinuric [9]. Urine data were not available for 3 dogs.

### Statistical Analysis

Statistical analysis was performed using a freeware statistical software package (Rv.3.4.3)*. Data were reported as percentage or frequency. Normal distribution of data was assessed using the Shapiro–Wilk normality test. Normally distributed data were reported as mean ± standard deviation (SD) and tested using the paired sample *t*-test, while non-normally distributed data were reported as median, minimum, and maximum and tested using the Wilcoxon signed-rank test. For all comparisons, the *p*-value was set at ≤0.05.

* Ref: The R Foundation for Statistical Computing, http://www.R-project.org (last accessed: 1 December 2020).

## 3. Results

The study included 22 client-owned dogs, subdivided as follows: 12 mixed-breed dogs (54.5%), 2 Brittany Spaniels (9.1%), 1 Australian Shepherd (4.5%), 1 Beagle (4.5%), 1 Dachshund (4.5%), 1 Deutscher Boxer (4.5%), 1 Golden Retriever (4.5%), 1 Maltese (4.5%), 1 Jack Russel terrier (4.5%), and 1 Pinscher (4.5%). Fourteen dogs were male (63.6%) and 8 were female (36.4%). The mean age was 10.7 ± 2.9 years and the mean body weight was 20.4 ± 10.2 kg. The primary lesion localization, the examined anatomical districts, and the CT scan provisional diagnosis can be found in Table 1.

At T0, all dogs received a single dose of iomeprol. The mean duration of anesthesia was 30.6 ± 4.8 min. Episodes of hypotension were not observed. The mean duration between T0 and T1 was 5.6 ± 1.3 days. Neither drugs nor fluids were administered between T0 and T1. Table 2 shows the mean and median values of hematological and urinary parameters at T0 and T1.

At T0, according to the IRIS staging system, 2 dogs (9.1%) were classified in CKD stage 2 [34]. An increase in SDMA concentration (27 µg/dL, 22 µg/dL, respectively) was observed in 2 dogs (9.1%), and an increase in serum total protein, sodium, potassium, and phosphorus concentrations was observed in 2 (9.1%), 1 (4.5%), 1 (4.5%), and 2 (9.1%) dogs, respectively. A decrease in serum total protein and albumin concentrations was observed in 2 (9.1%) and 9 (41%) dogs, respectively.

At T1, the serum creatinine concentration was within reference ranges in all dogs but one. In this dog, classified in CKD stage 2 at T0, the serum creatinine concentration did not change from T0 to T1. An increase in SDMA concentration was observed (16 µg/dL, 15 µg/dL, 25 µg/dL, 25 µg/dL, and 16 µg/dL, respectively) in 5 dogs (22.7%). Of these dogs, three (13.6%) presented normal SDMA at T0. Of the remaining 2 dogs, SDMA concentration changed from T0 to T1 (from 22 µg/dL to 25 µg/dL and from 27 µg/dL to 25 µg/dL, respectively). An increase in serum total protein, sodium, chloride, potassium, and phosphorus concentrations was observed in 3 (13.6%), 1 (4.5%), 1 (4.5%), 1 (4.5%), and 3 (13.6%) dogs, respectively; a decrease in serum total protein, albumin, and chloride concentrations was observed in 2 (9.1%), 10 (45%), and 1 (4.5%) dogs, respectively. Statistically significant differences in serum total protein, albumin, chloride, calcium, and phosphorus concentrations were found between T1 and T0 (Table 2).

Urinalysis was not available at T0 because of spontaneous micturition immediately before urinary collection and anesthesia for 3 dogs. For these dogs, urine collection was not performed at T1. No samples presented glycosuria. Hematuria was observed in 2 samples (10.5%) at T0. Rare to occasional transitional and/or squamous epithelial cells were observed in 14 samples (73.7%) at T0 and T1. Numerous transitional and squamous epithelial cells were observed in 4 samples (21%) at T0. Occasional granular casts were observed in 3 samples (15.8%) at T0 and 4 samples (21%) at T1, 3 of which were negative at T0. Pyuria was observed in 5 samples (26.3%) at T0 and 3 samples (15.8%) at T1, 1 of which was negative at T0. Bacteriuria was observed in 3 samples (15.8%) at T0 and 4 samples (21%) at T1, 2 of which were negative at T0. Urinary culture was not performed. Based on CT scan evaluation findings, benign prostate enlargement, prostatitis, and neoplasia of the bladder were suspected in 3 dogs presenting pyuria and/or bacteriuria. At T0, 2 (10.5%), 9 (47.4%), and 8 (42.1%) samples were classified as non-proteinuric, borderline proteinuric, and proteinuric, respectively. Among the 17 samples classified as borderline proteinuric and proteinuric, 11 (64.7%) were negative for pyuria and bacteriuria. Based on CT scan evaluation findings, benign prostate enlargement was suspected in 2 of these dogs. At T1, 5 (26.3%), 10 (5.3%), and 4 (21%) samples were classified as non-proteinuric, borderline proteinuric, and proteinuric, respectively. Among the 14 samples classified as borderline proteinuric and proteinuric, 10 (71.4%) were negative for pyuria and bacteriuria. Among these 10 samples, increased proteinuria from T0 to T1 was observed in 4 (40%) samples, one of which was non-proteinuric at T0. Results of urinalysis are summarized in Table 3. Statistically significant differences in USG and UPC were found between T1 and T0 (Table 2).

## 4. Discussion

This study evaluated for alterations in serum biochemical and urinary parameters before and within 1 week after IVIC administration during CT scanning under general anesthesia in a selected canine population.

Iomeprol belongs to second-generation low-osmolar, non-ionic monomers, iodinated compounds, which improves vascular tolerability and fewer side effects compared to those of first generation [35]. Various adverse reactions to IVIC administration in human beings have been reported [35,36,37]. On the contrary, there is a paucity of literature on alterations of serum biochemical and urinary parameters in dogs and adverse effects of contrast agents, in particular CI-AKI [20,28,30,38,39,40,41]. Partial decreases in glomerular filtration rate and AKI were described in some dogs following the injection of first-generation contrast agents, or higher doses of second-generation contrast agents [30,42,43]. In these studies, the criteria used to determine the glomerular filtration rate and to diagnose acute kidney injury were based on the determination of serum urea nitrogen and serum creatinine concentrations, endogenous creatinine clearance, and urine output. To date, only one experimental study has looked at CI-AKI in control dogs and dogs with gentamicin-induced AKI; the goal of that study was to investigate the effect of low-dose dopamine and 0.9% saline on CI-AKI and to improve excretory urogram image quality. Increased serum urea levels 72 h after IVIC administration were observed only in dogs with normal renal function, but not in those with impaired renal function [44], while a significant increase in the serum creatinine concentration was not observed in dogs of both groups. A possible protective effect of low-dose dopamine from CI-AKI development was hypothesized by the authors. More recently, a retrospective study of CI-AKI demonstrated a temporal association between the administration of IVIC and increases in serum creatinine concentration [31]. In this study, the median day of blood-work collection following IVIC administration was day 2, while peak increases in serum creatinine concentrations were seen 3–5 days following administration of contrast [8]. This may have contributed to decreased sensitivity for CI-AKI detection, or an underestimation of the severity of kidney injury. Although this relationship has not been previously established, the retrospective nature of the study makes it difficult to ascertain the true cause of renal injury in that population of patients. In our study, no difference in the median creatinine, BUN, and SDMA concentrations before and after IVIC administration was observed. In 5 dogs, an increase in SDMA concentration was observed at T1. Of these dogs, three presented normal SDMA at T0. SDMA, a relatively newly discovered renal biomarker, has been used successfully to diagnose acute and chronic kidney disease in dogs, but cannot be used to distinguish the two [45,46]. Based on a recent study, in non-azotemic dogs a cut-off of >18 µg/dL increases the specificity of SDMA for a glomerular filtration rate decrease of ≥40% without compromising sensitivity [47]. Consequently, for the 3 dogs of this study with SDMA concentrations <18 µg/dL, it is not possible to confirm a substantial decrease in glomerular filtration rate consistent with renal disease. In addition, dehydration could have contributed to SDMA changes. Indeed, if dehydration results in a prerenal azotemia reflecting a reduction in the glomerular filtration rate, then SDMA should also increase, along with serum albumin, total protein, urea, and creatinine concentrations [48]. In a study that included different types of human hematological malignancies, asymmetrical dimethylated arginine but not SDMA was shown to have significantly increased the concentration in the population with malignancies compared with the control group [49]. Similarly, in our oncologic population, it seems unlikely that an increased protein turnover led to an increase in SDMA. However, it cannot be confirmed but only hypothesized based on SDMA specificity for renal diseases in dogs [50,51].

In our study, serum albumin and total protein concentrations and USG were found to be significantly increased at T1. Hemoconcentration could explain these results, rather than contrast medium toxicity [52], while chronic inflammation or neoplasia could contribute to increases in total protein concentrations. In addition, an intraindividual variability among USG measurements cannot be excluded [53]. Recently, contrast media interference with routine chemistry results was investigated on less than 3-day-old serum samples [54]. Except for total protein and serum iron, for which values greater than cut-off values were observed, many of the analytes included in routine chemistry results were not significantly affected by iopamidol and iohexol. In our study, considering that the mean day of blood-work collection following IVIC administration was on day 5.6, it seems unlikely, although not demonstrated, that the increase in total serum protein concentration at T1 is caused by an interferential effect of the contrast medium. In a previous study, urinary excretion of IVIC caused significant alterations of USG 15 min after injection of IVIC in reference dogs and rendered urine samples unsuitable for diagnostic evaluation of renal tubular concentration capacity [55]. However, these results were obtained on urine samples collected shortly after IVIC administration; it seems therefore unlikely, although not demonstrated, that IVIC administration could have caused here an increased USG lasting several days.

A significant increase in serum calcium and phosphorus concentrations and a significant decrease in serum chloride concentration were found at T1. However, only serum phosphorus concentration varied outside the reference ranges. Serum phosphorus concentration shows poor sensitivity as it is thought that approximately 85% of the GFR must be lost before persistent hyperphosphatemia develops [56]. Since no dogs develop azotemia after IVIC administration, it seems unlikely that the increase in serum phosphorus concentration observed at T1 in some dogs could be related to decreased renal function. Since none of the 3 dogs that developed increased serum phosphorus concentration beyond the reference ranges had known osteolytic bone lesions, other causes not investigated at the time of our study or laboratory error should be considered. Overall, although there were changes in biochemistry parameters statistically significant between the time points, they were not clinically significant. Indeed, these changes were minimal and, except for phosphorus, a variation outside the reference ranges was not observed. Therefore, it is the authors’ opinion that these alterations are not indicative of a significant change in the underlying disease state in a <1 week period.

Urinalysis findings, such as glycosuria and proteinuria, can be indicative of renal injury [57]. Urine protein to creatinine ratio is useful to quantify urinary protein loss, once prerenal and post-renal causes have been excluded [58]. However, even in dogs with stable proteinuria, urine protein to creatinine ratio values may differ by as much as 40%, limiting its value in detecting transient nephropathy [58]. Excluding dogs with an active urine sediment, at T0, 11 samples were classified as borderline proteinuric and proteinuric, and at T1, 10 samples were classified as borderline proteinuric and proteinuric. Among these samples, increased proteinuria from T0 to T1 was observed in 4 samples, one of which was non-proteinuric at T0. Neoplasia is one of several known causes of proteinuria in dogs. Potential factors contributing to the development of proteinuria in dogs with cancer include decreased renal blood flow, injury induced by products of the tumor cells, and deposition of antigen–antibody immune complexes [59,60,61]. The degree of proteinuria is generally mild, typically requiring monitoring rather than immediate intervention [59]. In addition, 25% to 31% of apparently healthy middle age to geriatric dogs are persistently proteinuric [62]. Based on these observations, proteinuria associated with old age or neoplasia seems plausible for most of our dogs. However, other causes of proteinuria should also be considered. Medications, most notably corticosteroids and toceranib phosphate, have been shown to induce proteinuria in dogs [63,64]. As previously reported, none of the dogs in this study received prednisone, prednisolone, or toceranib between T0 and T1. Moreover, the contributory role of comorbidities such as infectious diseases, systemic inflammatory diseases, and systemic hypertension cannot be ruled out here without more exhaustive testing [65]. Finally, acute and chronic renal diseases have been associated with proteinuria in dogs [66]. The International Renal Interest Society classifies persistent, non-azotemic renal proteinuria as stage 1 chronic kidney disease [34]. Since at T0, except for the 2 dogs classified in the CKD stage 2 group, azotemia was not observed, it cannot be excluded that some dogs with proteinuria at T0 fall into IRIS stage 1 group. With regard to the non-proteinuric dog at T0 that developed borderline proteinuria after IVIC administration, it is unknown whether one of the aforementioned causes or contrast administration itself played a role. However, since the dog’s urine protein to creatinine ratio value changed by only 14% from T0 to T1, it is not possible to confidently assume that the magnitude of proteinuria has actually changed.

Measurement of the fractional excretion of electrolytes would have been an additional interesting tool for the investigation of kidney function in our dogs [67,68]. Since the determination of the fractional excretion requires the collection of urine over a long period of time, the excretion ratio has been recently suggested as a surrogate marker to follow trends in solute excretion [69]. However, the diagnostic value of fractional excretion in dogs needs to be further explored and cannot be routinely recommended because of the possible misinterpretation of renal tubular dysfunction [67].

Specific enzymes (γ-glutamyl transferase or GGT, N-acetyl-β-glucosaminidase or NAG, and alkaline phosphatase or ALP) can be used as markers for renal tubular injury, since their urinary activity is primarily of tubular origin [70,71]. Moreover, uGGT and uGGT/uc can be used to detect dogs with early, non-azotemic AKI [72,73]. However, detection of AKI might be influenced by the timing of their measurement and, beyond aminoglycoside induced AKI, information of urinary enzyme activity in dogs with naturally occurring AKI is scarce [74]. Several in vitro and in vivo factors affect uALP and uGGT activity. Pyuria, hematuria, and being an intact male may interfere with uGGT and uALP measurement [32], which may partly account for increased uGGT and uALP activities in some dogs of our population. In addition, normalization to urine creatinine entails several limitations, including larger inter-individual urine creatinine excretion variations, even in healthy dogs [74]. In our study, no significant differences were found in uALP, uGGT, uALP/uc, and uGGT/uc before and after IVIC administration. Moreover, the mean values of uALP, uGGT and uGGT/uc seem to be close to the values reported for healthy animals or animals without acute kidney injury [72,73,75], although established reference ranges are lacking.

The evaluation of other early biomarkers for acute kidney injury such as neutrophil-gelatinase-associated lipocalin and kidney injury molecule-1 would have been of potential benefit [72,76]. However, the specificity of neutrophil–gelatinase-associated lipocalin is affected by systemic inflammation [77], including neoplasia, while kidney injury molecule-1 levels have been shown to be higher in patients with acute tubular necrosis, compared to those with contrast-induced nephropathy [78].

The main limitation of the present study is that imaging was performed for different types of pathologies, thus making it difficult to interpret some results at T0, such as proteinuria, as previously discussed. However, since no therapies were implemented in this 1 week period, it is unlikely that a significant change in the underlying state of most of the dogs influenced the results in a <1 week period. The limited number of cases included in our study, which is due to the low incidence of CI-AKI [31], might cause a further limitation. However, since the design of this study did not skew the population toward a sicker group of patients, the results presented here may be reliable. The third possible limitation is the timing of T1 without a serial collection of serum and urine samples in the interim. While this design did not allow us to determine whether there were changes in the more acute period [79], it does indicate that there were no clinically significant alterations after several days. A further plausible limitation is the collection method that may have altered urine analysis results. Indeed, voided samples may contain environmental and lower urinary tract contaminants, red and white cells, and bacteria from the distal urethra or genital tracts which may affect USG, UPC, and sediment examination. Despite the above-mentioned limitations, this method is time-saving and does not require a skilled person to collect urine sample. Another limitation is represented by the effect of anesthetic drugs, fluid therapy during the procedure, and length of fasting prior to both sample collections. However, since similar anesthetic and IV fluid protocols were used and a 12 h fasting period was required, it is the authors’ opinion that the influence of these factors on the results was minimal. A further consideration is that the T1 collection did not occur shortly after anesthesia and fluid therapy, which might have plausibly altered the results. Finally, a control group was not available for comparison. However, it should be noted that the demonstration of a causal association between clinicopathologic alterations and IVIC administration, for which a control group would have been mandatory, was beyond the scope of this study.

## 5. Conclusions

In brief, only changes of minimal clinical relevance in the measured serum biochemical and urinary parameters were detected within 1 week of administration of IVIC compared to baseline. Further studies are suggested to help to further assess potential effects of IVIC in other dog populations.

## Figures and Tables

**Table 1:** Primary lesion localization, examined anatomical districts, and the computed tomography (CT) scan provisional diagnosis of 22 dogs.

Primary Lesion Localization	Examined Anatomical District	CT Scan Provisional Diagnosis
R caudal and M lung lobes	T, A, P	Primary pulmonary neoplasia
R adrenal gland	T, A, P	R adrenal gland neoplasia, CVV thrombosis
Lymph nodes	T, A, P	Abdominal and thoracic LP
R parotid gland	TB	R parotid gland neoplasia, R mandibular and retropharyngeal LP
Prostate gland	TB	Prostate neoplasia, iliac and sacral metastatic LP
Parasternal L chest wall	T, A	L parasternal neoplasia, pulmonary neoplasia, L axillary LP
L thigh region	T, A, P	L thigh subcutaneous neoplasia, L popliteal LP
Nasal cavities, frontal region	TB	Frontal neoplasia
L axillary lymph node	T	L axillary LP, R cranial lung lobe neoplasia
Infraorbital region	TB	L infraorbital neoplasia
Bladder	T, A, P	Bladder neoplasia
R humerus region	T, A, P	Sternal neoplasia
L jaw	TB	L mandibular neoplasia, L retropharyngeal LP
R inguinal mammary gland	T, A, P	No abnormal findings
Nasal cavities and sinuses, nasal planum	TB	Nasal planum neoplasia, R mandibular LP
Spleen	T, A, P	Splenic neoplasia
R axilla	TB	R axillary region neoplasia, chronic prostatitis
L posterior cranial fossa	TB	L posterior cranial fossa neoplasia, BPE
L middle ear	Head	L otitis media
Rostral maxilla	TB	Rostral maxilla neoplasia
Liver	TB	R lateral lobe cysts, BPE
R apical lung lobe	TB	Primary pulmonary neoplasia, BPE

A = abdomen; BPE = benign prostate enlargement; CT = computed tomography; CVV = caudal vena cava; L = left; LP = lymphadenopathy, M = middle; P = pelvis; R = right; T = thorax; TB = total body.

**Table 2:** Results of selected biochemical and urinary parameters of 22 dogs before (T0) and after (T1) IVIC administration.

Parameter	T0	T1	Reference Ranges	*p*-Value
Creatinine (mg/dL)	0.8 (0.4–1.7)	0.8 (0.4–1.7)	0.5–1.5	0.5
BUN (mg/dL)	13 (8–56)	15 (9–57)	9–29	0.7
SDMA (µg/dL)	10 (7–27)	10 (8–25)	0–14	0.2
Total protein (g/L)	63.5 (±7.3)	66.5 (±7.5)	54–76	0.004 *
Albumin (g/L)	26.9 (±3.9)	27.7 (±4.1)	28–43	0.047 *
Sodium (mmol/L)	148 (144–155)	148 (145–158)	142–153	1
Chloride (mmol/L)	113 (110–119)	112 (105–122)	106–120	0.041 *
Potassium (mmol/L)	4.6 (4–6.3)	4.7 (4–6)	3.9–5.8	0.2
Calcium (mmol/L)	2.4 (±0.2)	2.5 (±0.1)	2.1–2.9	0.002 *
Phosphorus (mmol/L)	1.2 (0.9–1.8)	1.4 (0.9–2.4)	0.9–1.7	0.029 *
USG	1.025 (±10.9)	1.030 (±11.4)	1.015–1.045	0.019 *
UPC	0.5 (0.1–6.3)	0.2 (0.1–2.3)	<0.2	0.016 *
uALP/uc (U/L)/(g/L)	9.1 (0.9–1886.8)	6.3 (1.4–20.3)	0.25 ± 1.17 U/g; [32]	0.1
uALP (U/L)	9.6 (2.6–867.9)	8.4 (2.6–32.6)	0–55 U/L; [33]	0.3
uGGT/uc (U/L)/(g/L)	33.6 (11.9–522.2)	35.5 (15.1–392.9)	<42 U/g [32]	0.6
uGGT (U/L)	48.3 (11.4–489.8)	53.1 (9.6–843.2)	6–112 U/L [32]	0.4

Data are reported as mean (±SD) or median (minimum-maximum) based on their distribution. * Statistically significant (*p*-value ≤ 0.05); BUN = blood urea nitrogen; IVIC = intravenous iodinated contrast medium; SDMA = symmetric dimethylarginine; uALP = urinary alkaline phosphatase; uALP/uc = urinary alkaline phosphatase to urinary creatinine ratio; uGGT = urinary γ-glutamyl transpeptidase; uGGT/uc = urinary γ-glutamyl transpeptidase to urinary creatinine ratio; UPC = urine protein to creatinine ratio; USG = urine specific gravity. Reference ranges for uALP/uc, uALP, uGGT/uc, uGGT have been extrapolated from the references [32] and [33].

**Table 3:** Results of urinalysis in dogs before (T0) and after (T1) IVIC administration *.

	Glycosuria n	Hematuria n	Epithelial Cells n	Casts n	Non-Proteinuric n	Borderline Proteinuric n	Proteinuric n	UPC ≥ 0.2 n
T0	0	1	12	2	2	6	5	11
T1	0	0	11	3	4	7	3	10

* = data of dogs with post-renal proteinuria are not shown. n = number of samples; IVIC = intravenous iodinated contrast medium; UPC = urine protein to creatinine ratio.

## Data Availability

All data analyzed during this study are included in this published article.
